# Primary Sjögren's syndrome: Longitudinal real‐world, observational data on health‐related quality of life

**DOI:** 10.1111/joim.13451

**Published:** 2022-01-24

**Authors:** Jessica Tarn, Dennis Lendrem, Peter McMeekin, Clare Lendrem, Ben Hargreaves, Wan‐Fai Ng

**Affiliations:** ^1^ Translational and Clinical Research Institute Newcastle University Newcastle upon Tyne UK; ^2^ Faculty of Health and Life Science Northumbria University Newcastle upon Tyne UK; ^3^ NIHR Newcastle In Vitro Diagnostics Co‐operative Newcastle University Newcastle upon Tyne UK; ^4^ Musculoskeletal Directorate Newcastle‐upon‐Tyne Hospitals NHS Foundation Trust Newcastle upon Tyne UK

**Keywords:** health‐related quality of life, longitudinal, Sjogren's

## Abstract

**Introduction:**

Primary Sjögren's syndrome (pSS) is a chronic inflammatory condition, which presents with symptoms of dryness, pain, fatigue and often symptoms of anxiety and depression. Health‐related quality of life (HRQoL) is significantly reduced in pSS and the direct and indirect health costs of pSS are substantial. This study aims to determine how symptom burden, disease activity and demographics associate with HRQoL longitudinally over a median of 24‐month follow‐up period in pSS.

**Methods:**

Longitudinal EuroQoL‐5 dimension (EQ‐5D)–3L data from the Newcastle pSS cohort (*n* = 377) were evaluated using a survival analysis strategy. Kaplan–Meier and Cox proportional hazards analysis were performed using baseline Newcastle Sjogren's Stratification Tool (NSST) subgroup, EULAR Sjogren's Syndrome Patient Reported Index (ESSPRI), EULAR Sjogren's Syndrome Disease Activity Index (ESSDAI), disease duration, age and sex as covariates including polypharmacy and comorbidity score, where data were available (*n* = 191).

**Results:**

Of the 377 pSS participants analysed in this study, 16% experienced a decline in HRQoL to a health state comparable to or worse than death. NSST subgroup and ESSPRI score had a significant relationship with time to ‘EQ‐5D event’, whereas baseline ESSDAI, age, disease duration and sex did not.

**Conclusion:**

In pSS, symptom burden and to a great extent NSST subgroup, rather than systemic disease activity, has a significant relationship with HRQoL longitudinally. Improvements in symptom burden have the potential to produce significant impacts on long‐term HRQoL in pSS.

## Introduction

Primary Sjögren's syndrome (pSS) is a chronic inflammatory condition that commonly presents with symptoms of dry eyes, dry mouth, pain and fatigue. Symptoms of anxiety and depression are also common. There is currently no effective treatment for pSS and the direct and indirect health costs of pSS are substantial.

Health‐related quality of life (HRQoL) estimates are a key contributor to health economic evaluations. Cost–utility analyses for new treatments are assessed in quality‐adjusted life‐years (QALYs), which combine HRQoL and survival [1]. EuroQoL‐5 dimension (EQ‐5D) is a standardised instrument for estimating HRQoL on five different dimensions: mobility, self‐care, usual activities, pain/discomfort and anxiety/depression. The score is mapped to a single utility value or ‘EQ‐5D score’ using reference data from the UK population provided by the EuroQoL group (http://www.euroqol.org). Scores 0–1 represent death and full health, respectively, whereas scores below zero indicate health states 'worse than death'. EQ‐5D is a generic utility instrument and can be compared across diseases. EQ‐5D is also often used as a key metric in cost–utility analysis in therapeutic development.

In pSS, HRQoL is significantly reduced [2]. The most important correlates of EQ‐5D UK utility are pain/discomfort and anxiety/depression [2, 3]. However, it is not known how disease activity and symptom burden affect HRQoL longitudinally. Recently we identified distinct subgroups within the UK Primary Sjogren's Registry (UKPSSR) cohort, demonstrated biological differences between those subgroups and developed a stratification tool (Newcastle Sjogren's Stratification Tool [NSST]) [4]. NSST uses patient‐reported symptom scores for dryness, fatigue, pain, anxiety and depression to assign pSS sufferers into four symptom burden–based subgroups, namely the high symptom burden (HSB), low symptom burden (LSB), dryness‐dominant fatigue (DDF) and pain‐dominant fatigue (PDF) subgroups. Patients in the HSB subgroup report high scores for all five symptoms, LSB group patients report low scores for all symptoms, PDF group patients report high scores for dryness, pain and fatigue and DDF group patients report high scores for dryness and fatigue. In this study, we explore the relationship between the NSST subgroup, symptom burden, disease activity and the trajectory of HRQoL in pSS.

## Methods

Research ethics approval was granted by the UK North‐West Research Ethics Committee.

### Subjects

All longitudinal data were collected from a regional Sjogren's clinic based in Newcastle‐upon‐Tyne between August 2009 and December 2020 (*n* = 507). All participants fulfil the American European Consensus Group classification criteria [5]. Data were collected prospectively using standardised proforma at the time of clinic appointment. For all patients attending the regional Sjogren's clinic, the following data are collected as part of their routine care: EULAR Sjögren's Syndrome Disease Activity Index (ESSDAI) [6], EULAR Sjögren's Syndrome Patient Reported Index (ESSPRI) [7], Hospital Anxiety and Depression Scale (HADS) [8], EQ‐5D UK utility and Profile of Fatigue (Pro‐F) [9]. Follow‐up data were available for 377 subjects (Table 1) with a median follow‐up time of 2.1 years. Patients who had not yet attended their follow‐up appointment at the time of analysis were not included. Baseline polypharmacy and comorbidity data were available from the UKPSSR (http://www.sjogrensregistry.org) for a proportion of participants (*n* = 191). The following variables were available at baseline for all subjects: age, disease duration, sex, Ro/La status, ESSDAI, ESSPRI, HADS, EQ‐5D UK utility and Pro‐F.

**Table 1:** Clinical and demographic characteristics of the cohort at baseline visit. Values shown are median values with (25th and 75th) percentiles

Clinical characteristics (*n* = 377)	Baseline	
Age	57 (49–67)	
Disease duration (AECG years)	6.7 (2–8)	
BMI	26.0 (23.4–29.8)	
Sex	331 F, 46 M	
Ro/La positivity	123 negative, 253 positive	
Symptom burden group	DDF 68, HSB 99, LSB 41, PDF 169	
Follow‐up time (years)	2.1 (1.2–5.1)	
Total number of follow‐up appointments	3 (2, 4)	

	Baseline	Follow‐up
ESSDAI score	2.0 (0.0–4.0)	1.0 (0.0–3.0)
ESSPRI score	6.0 (4.6–7.6)	6.3 (4.7–7.6)
EQ‐5D score	0.69 (0.4–0.8)	0.69 (0.2–0.8)

BMI, body mass index; F, female; M, male; DDF, dryness dominant fatigue; HSB, high symptom burden; LSB, low symptom burden; PDF, pain dominant fatigue; ESSDAI, EULAR Sjogren's syndrome disease activity index; ESSPRI, EULAR Sjogren's syndrome patient reported index.

### Polypharmacy and comorbidity score

Polypharmacy score was calculated as the sum of medications at the time of clinic appointment. Details of comorbid conditions were collected prospectively, standardised and converted to clinically meaningful categories using Clinical Classifications Software Refined (CCSR) tools [10]. A Chronic Condition Indicator score (CCI) was calculated using CCSR tools, which was used in risk analyses.

### Data analysis

Longitudinal health outcome data (EQ‐5D UK utility) were analysed. As the data were collected during routine clinic appointments, the time between visits and total number of visits differed between patients. The EQ‐5D UK utility scores were calculated as previously described [2] and evaluated using a survival analysis strategy. As EQ‐5D UK utility scores can represent the quality of life at death or worse than death, we used an EQ‐5D UK utility threshold of greater than zero to represent ‘survival’. The follow‐up time at which EQ‐5D UK utility fell to zero or below was recorded as an ‘EQ‐5D event’. Time to event analysis was performed using baseline NSST subgroup, ESSPRI, ESSDAI, disease duration, age, sex, polypharmacy score and comorbidity score as covariates. Kaplan–Meier estimator curves and Cox proportional hazards analysis were performed in R.

### Patient and public involvement

Patients were involved in the planning and conduct of this research and have identified priority areas for pSS research that are most relevant to their experience of the disease, which include fatigue and general well‐being. Patient partner representatives are included in the project steering committee.

## Results

The distribution of EQ‐5D scores in this pSS cohort is bimodal with a peak around 0 and another around 0.75; there is possibly a third peak of patients who report an EQ‐5D score of 1, perfect health. Of the 377 pSS participants analysed in this study, 69 (18%) experienced a low (<0.5) EQ‐5D utility score and 59 (16%) experienced a decline in quality of life to a health state comparable to or worse than death.

**Fig. 1:**
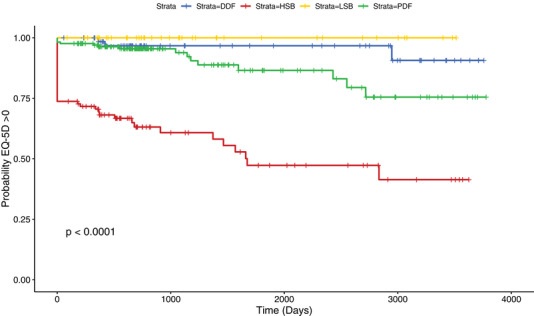
Kaplan–Meier estimator curves showing time to ‘EQ‐5D event’ data between four symptom burden groups. ‘EQ‐5D event’ = EQ‐5D ≤0. Strata = NSST subgroup, *n* = 377. EQ‐5D, EuroQoL‐5 dimension.

NSST subgroup at baseline had a significant relationship with time to threshold EQ‐5D event (*p* = <0.001) (Fig. 1). Increased risk of an ‘EQ‐5D event’ was most highly associated with membership of the HSB subgroup, defined by an overall HSB at baseline (Fig. 1). No members of the LSB subgroup reported an EQ‐5D of zero or below and the DDF subgroup reported very few ‘EQ‐5D events’ (*n* = 3, 5%). In the PDF and HSB groups, the proportions of ‘EQ‐5D events’ are 15% (*n* = 15) and 41% (*n* = 41), respectively. The median EQ5D utility values at baseline and time of event or end of follow‐up are 0.85 and 0.80, respectively, for the LSB group and 0.74 and 0.69, respectively, for the DDF group; whereas the median EQ5D utility values at baseline and time of event or end of follow‐up are 0.52 and 0.19, respectively, for the HSB group and 0.69 and 0.68, respectively, for the PDF group. See Table S1 for further details.

**Fig. 2:**
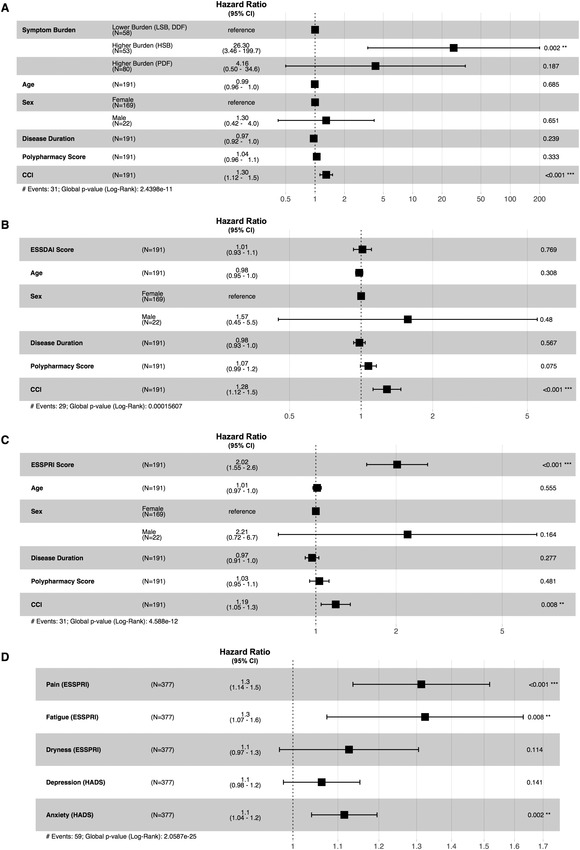
Graphical summaries of four Cox proportional hazards models (a–d). The estimated HR, 95% CI of the HR and p‐value are shown for each covariate in each model. The models show the impact of (a) the NSST subgroup, (b) disease activity (ESSDAI) and (c) ESSPRI, on risk of ‘EQ5D event’ occurrence using demographic covariates age, sex, disease duration, polypharmacy score and CCI. (d) This model includes the individual symptom scores used in NSST stratification. CCI, Chronic Condition Indicator ; CI, confidence interval; DDF, dryness dominant with fatigue; HADS, Hospital Anxiety and Depression Scale; HR, hazard ratio; HSB, high symptom burden; LSB, low symptom burden; PDF, pain dominant with fatigue.

Analysis of Cox proportional hazards identified the NSST subgroup as the most significant ‘risk factor’ for an ‘EQ‐5D event’ (Fig. 2 a). The HSB subgroup was associated with the highest hazard ratio (HR) (estimated HR = 26.30, confidence interval [CI] = 3.5–199.7, *p* = 0.002). This effect was consistent after correction for polypharmacy score and CCI. CCI has a close relationship with polypharmacy score and is associated with a small but significant (estimated HR = 1.30, CI = 1.1–1.5, *p* < 0.001) risk of reduced EQ‐5D. Baseline ESSDAI, age at baseline visit, disease duration and sex were not significant risk factors for an ‘EQ‐5D event’ (Fig. 2 b). High ESSPRI score (Fig. 2 c) is a significant risk factor for a decline in EQ‐5D (estimated HR = 2.02, 1.6–2.6, *p* < 0.001). NSST strata are calculated using five patient‐reported symptom scores. The impact of each score at baseline (Fig. 2 d) indicates that symptoms of pain, fatigue and anxiety contribute most significantly to the risk of ‘EQ‐5D event’, although the HRs of individual symptom are relatively low (estimated HRs = 1.30, 1.30 and 1.10, CIs = 1.1–1.5, 1.1–1.6 and 1.0–1.2, respectively).

## Discussion

In pSS, patients have significantly lower health utility values compared to the UK general population, which is confirmed in this study [2]. The median EQ‐5D utility value in this cohort is lower than the UK population norm for this age group [11]. Using longitudinal data, we observe that in a small proportion of the cohort, HRQoL declines significantly in a relatively short time frame. The membership of an HSB subgroup has a significant relationship with HRQoL longitudinally even after adjustment for other factors associated with quality‐of‐life decline such as age, polypharmacy and chronic comorbid conditions. LSB and DDF groups have very good quality of life outcomes longitudinally whereas PDF and especially HSB groups show reduced quality of life at baseline and further decline during the study period. Heterogeneity in the range and severity of key symptoms of pSS poses a challenge for patient care, experimental design and therapeutic development [4, 12]. Classification of patients using patient‐reported scores is a promising new approach to stratified medicine. The NSST is an easily applicable method for patient stratification in large cohorts using symptom burden scores. Our observation that the NSST subgroup is the strongest predictor for EQ‐5D UK utility decline underscores the value of the NSST stratification tool in clinical management and therapeutic development. For instance, our data support the development of an effective clinical strategy to manage symptoms such as pain, fatigue and low mood in the HSB and PDF subgroups, which likely requires a personalised, holistic approach [13]. In chronic conditions such as pSS, improving the HRQoL is critical, and HRQoL and health utility are key considerations in therapeutic development especially in public‐funded healthcare systems. Therefore, we would advocate cautions in the inclusion of the LSB group in trials of novel therapies as there is very little potential improvement in terms of HRQoL and QALY gains for this group. In contrast, targeting treatments to the HSB and PDF subgroups may lead to more cost‐effective treatment strategies.

The individual domains that comprise the assignment of NSST subgroups are derived from the individual ESSPRI domains and HADS anxiety and depression subscores. The total ESSPRI Score, fatigue, pain and to a small extent anxiety are significantly related to EQ‐5D UK utility decline. Importantly, this mirrors the key concerns highlighted by patient partner representatives.

In this study, clinical measures of disease activity in pSS, such as ESSDAI, are poor predictors of EQ‐5D UK utility decline. The median ESSDAI score in this cohort is relatively low, which may potentially impact the ability to demonstrate any significant relationship between ESSDAI and EQ‐5D utility value over time. Nonetheless, our findings call for caution in using ESSDAI as the main clinical endpoint to assess the therapeutic efficacy of new therapies.

The limitations of this study are that the data are observational from a single regional centre; therefore, independent validation using international cohorts is necessary. It is important to also note that EQ‐5D UK utility may not represent the impact of all aspects of pSS on HRQoL; therefore, other assessment tools should be also be explored, for example ICECAP‐A [14].

## Conflict of interest

None of the authors has competing interests related to the submitted work. Wan‐Fai Ng reports personal fees from GlaxoSmithKline, MedImmune, Novartis and BMS; personal fees and grant support from Abbvie and grant support from Resolves Therapeutics, Nascient, outside of the submitted work.

## Funding statement

This work was supported by Foundation of Research in Rheumatology (FOREUM): (grant number: 022) and Medical Research Council, UK funding (grant number: G0800629). This study also received infrastructure support from the NIHR Newcastle Biomedical Research Centre and the NIHR Newcastle Clinical Research Facility.

## Author contributions

Jessica Tarn: conceptualization; formal analysis; writing – original draft; writing – review and editing. Dennis Lendrem: formal analysis; writing – review and editing. Peter McMeekin: formal analysis; writing – review and editing. Clare Lendrem: data analysis. Ben Hargreaves: data curation. Wan‐Fai Ng: funding acquisition; supervision; writing – review and editing. Wan‐Fai Ng: conceptualisation, funding acquisition, data interpretation, writing – review and editing.

## Supporting information


**Figure S1**. Histogram of EQ‐5D UK utility distribution showing 3 groupings of patients centered around EQ‐5D UK utility scores of 0, 0.6 and 1. A, First Visit; B, Last Visit; C, Baseline values are shown in teal with end of follow up values overlaid in red.

## References

[1] Higgins AM , Harris AH . Health economic methods: cost‐minimization, cost‐effectiveness, cost‐utility, and cost‐benefit evaluations. Crit Care Clin. 2012;28(1):11–24. 10.1016/j.ccc.2011.10.002 22123096

[2] Lendrem D , Mitchell S , McMeekin P , Bowman S , Price E , Pease CT , et al. Health‐related utility values of patients with primary Sjögren's syndrome and its predictors. Ann Rheum Dis. 2014;73(7):1362–8. 10.1136/annrheumdis-2012-202863 23761688

[3] Lendrem D , Mitchell S , McMeekin P , Gompels L , Hackett K , Bowman S , et al. Do the EULAR Sjögren's syndrome outcome measures correlate with health status in primary Sjögren's syndrome? Rheumatology. 2015;54(4):655–9. 10.1093/rheumatology/keu361 25240612

[4] Tarn JR , Howard‐Tripp N , Lendrem DW , Mariette X , Saraux A , Devauchelle‐Pensec V , et al. Symptom‐based stratification of patients with primary Sjögren's syndrome: multi‐dimensional characterisation of international observational cohorts and reanalyses of randomised clinical trials. Lancet Rheumatology. 2019;1(2):e85–94. 10.1016/S2665-9913(19)30042-6 PMC7134527 38229348

[5] Vitali C , Bombardieri S , Jonsson R , Moutsopoulos HM , Alexander EL , Carsons SE , et al. Classification criteria for Sjögren's syndrome: a revised version of the European criteria proposed by the American–European Consensus Group. Ann Rheum Dis. 2002;61:554–8. 10.1136/ard.61.6.554 12006334 PMC1754137

[6] Seror R , Ravaud P , Bowman SJ , Baron G , Tzioufas A , Theander E , et al. EULAR Sjogren's Syndrome Disease Activity Index: development of a consensus systemic disease activity index for primary Sjogren's syndrome. Ann Rheum Dis. 2010;69:1103–9. 10.1136/ard.2009.110619 19561361 PMC2937022

[7] Seror R , Ravaud P , Mariette X , Bootsma H , Theander E , Hansen A , et al. EULAR Sjogren's Syndrome Patient Reported Index (ESSPRI): development of a consensus patient index for primary Sjogren's syndrome. Ann Rheum Dis. 2011;70:968–72. 10.1136/ard.2010.143743 21345815

[8] Zigmond AS , Snaith RP . The Hospital Anxiety And Depression Scale. Acta Psychiatr Scand. 1983;67(6):361–70. 10.1111/j.1600-0447.1983.tb09716.x 6880820

[9] Bowman SJ , Booth DA , Platts RG , UK Sjögren's Interest Group . Measurement of fatigue and discomfort in primary Sjogren's syndrome using a new questionnaire tool. Rheumatology. 2004;43(6):758–64. 10.1093/rheumatology/keh170 15039495

[10] Clinical Classifications Software Refined (CCSR) . Healthcare Cost and Utilization Project (HCUP). October 2021. Agency for Healthcare Research and Quality, Rockville, MD. www.hcup-us.ahrq.gov/toolssoftware/ccsr/ccs_refined.jsp. Last modified: 28 Oct 2021. Accessed May 21, 2021.

[11] Janssen B , Szende A . Population norms for the EQ‐5D. In: Szende A , Janssen B , Cabases J , editors. Self‐reported population health: an international perspective based on EQ‐5D. Dordrecht: Springer; 2014. p. 19–30. 10.1007/978-94-007-7596-1_3 29787044

[12] Gandolfo S , De Vita S . Emerging drugs for primary Sjögren's syndrome. Expert Opin Emerg Drugs. 2019;24(2):121–32. 10.1080/14728214.2019.1634052 31286787

[13] Davies K , Dures E , Ng W‐F . Fatigue in inflammatory rheumatic diseases: current knowledge and areas for future research. Nat Rev Rheumatol. 2021;17(11):651–64. 10.1038/s41584-021-00692-1 34599320

[14] Al‐Janabi H , Flynn TN , Coast J . Development of a self‐report measure of capability wellbeing for adults: the ICECAP‐A. Qual Life Res. 2012;21(1):167–76. 10.1007/s11136-011-9927-2 21598064 PMC3254872

