# Supplementary material for: Primary Sjögren's syndrome: Longitudinal real‐world, observational data on health‐related quality of life
Source: J Intern Med. 2022 Jan 24;291(6):849–55. doi: 10.1111/joim.13451 (PMC9305875; doi:10.1111/joim.13451)
Supplement: Supplementary file 1 — Figure S1. Histogram of EQ‐5D UK utility distribution showing 3 groupings of patients centered around EQ‐5D UK utility scores of 0, 0.6 and 1. A, First Visit; B, Last Visit; C, Baseline values are shown in teal with end of follow up values overlaid in red. [file JOIM-291-849-s001.docx]

Supplementary Materials

A


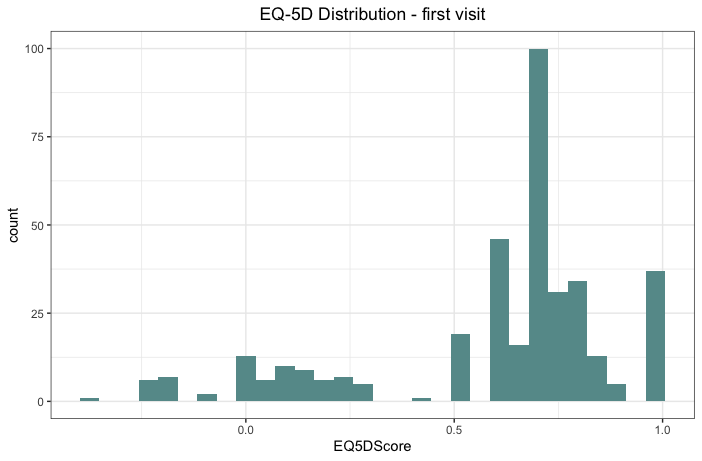


B

C
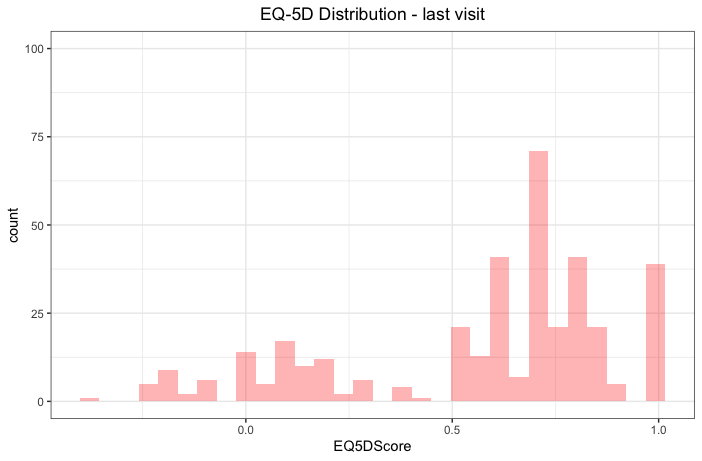

Supplementary Figure S1. Histogram of EQ-5D UK utility distribution showing 3 groupings of patients centered around EQ-5D UK utility scores of 0, 0.6 and 1. A, First Visit; B, Last Visit; C, Baseline values are shown in teal with end of follow up values overlaid in red.

|  |  | **EQ-5D UK Utility** | | |
| --- | --- | --- | --- | --- |
| **Strata** | **Time Point** | **median** | **quantile 25%** | **quantile 75%** |
| DDF | Baseline | 0.74 | 0.69 | 0.85 |
|  | Time of event / End of Follow up | 0.69 | 0.62 | 0.85 |
| HSB | Baseline | 0.52 | -0.02 | 0.66 |
|  | Time of event / End of Follow up | 0.19 | -0.07 | 0.64 |
| LSB | Baseline | 0.85 | 0.73 | 1.00 |
|  | Time of event / End of Follow up | 0.80 | 0.73 | 1.00 |
| PDF | Baseline | 0.69 | 0.62 | 0.73 |
|  | Time of event / End of Follow up | 0.69 | 0.52 | 0.76 |
